# Developing pressures: fluid forces driving morphogenesis

**DOI:** 10.1016/j.gde.2015.01.010

**Published:** 2015-02-17

**Authors:** Adam Navis, Michel Bagnat

**Affiliations:** a Department of Cell Biology, Duke University Medical Center, 333B Nanaline Duke Bldg., Box 3709, Durham, NC, USA

## Abstract

Over several decades genetic studies have unraveled many molecular mechanisms that underlie the signaling networks guiding morphogenesis, but the mechanical forces at work remain much less well understood. Accumulation of fluid within a luminal space can generate outward hydrostatic pressure capable of shaping morphogenesis at several scales, ranging from individual organs to the entire vertebrate body-plan. Here, we focus on recent work that uncovered mechanical roles for fluid secretion during morphogenesis. Identifying the roles and regulation of fluid secretion will be instrumental for understanding the mechanics of morphogenesis as well as many human diseases of complex genetic and environmental origin including secretory diarrheas and scoliosis.

## Introduction

The generation of a complete body-plan from a single cell during embryonic development depends on complex interactions between molecular signals and morphogenetic forces. Genetic analyses have identified key molecular mechanisms controlling morphogenesis, but how molecular functions translate to specific morphogenetic movements is still poorly understood. Research into the mechanics of development has been focused largely on short range forces generated at the cell cortex by actin contractility. However, it has become increasingly clear that more broadly acting forces like fluid flow and hydrostatic pressure play substantial roles during morphogenesis. These “fluid forces” are particularly important during tubulogenesis, a key process underlying the structural organization of most organs. Here, we review the role of hydrostatic pressure during morphogenesis in metazoans; from tubulogenesis to axis elongation and spine formation, and the pathological implications of misregulated fluid secretion.

## Fluid secretion 101

**Figure 1:**
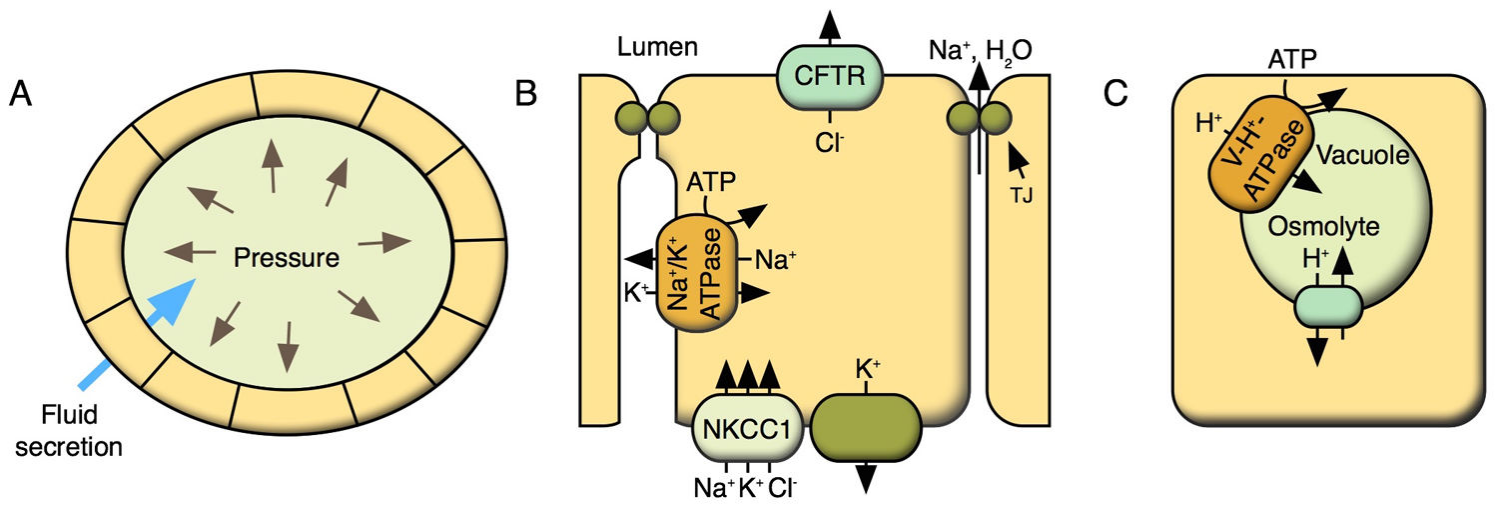
Mechanics of fluid secretion (A) Fluid accumulation within an enclosed area generates an outward pressure capable of inflating a luminal space. (B) Schematic representation of a simplified model of fluid secretion across epithelia through coordinated activity of ion channels that establish electrochemical and osmotic gradients. These gradients drive water into the lumen. (C) The V-H^+^ -ATPase powers H^+^ transport into a vacuolar space, which helps transport an unknown osmolyte into the lumen to draw water into the organelle.

The control of water transport is an essential physiological function for all organisms. Even though water accounts for most of the mass and volume of an organism, cells are unable to directly control its movement. Instead, they rely on ionic and osmotic gradients to power fluid transport. The incompressible nature of liquids allows fluid secreted into luminal spaces to generate considerable hydrostatic pressure that is capable of acting at great distances (Figure 1A).

The osmotic gradients that drive fluid secretion across epithelial cells are powered by ATP hydrolysis by the Na^+^/K^+^-ATPase, which generates a sodium gradient [1-5]. The extracellular sodium is then used to import chloride through symporters like NKCC1. Chloride can then be transported into the lumen through channels like CFTR, bestrophins, or TMEM16a to generate a luminal electrochemical gradient, which draws sodium ands water into the lumen (Figure 1B). Fluid can also accumulate within intracellular organelles powered by H^+^ transport through the V-H^+^-ATPase (Figure 1C).

Fluid secretion across epithelia also depends on the paracellular barrier formed by membrane proteins of the claudin (cldn) and occludin families [6]. These proteins interact across cell boundaries and also laterally within the same cell forming the tight junction (TJ) arrays, which can form ion-selective pores that help regulate fluid secretion [7] (Figure 1B).

## The organogenesis bubble: opening and resolution of the lumen

Tubes are a basic structural element underlying the morphology of most organs. Although the specific morphogenetic strategies may vary between organs and across species, tubulogenesis invariably presents two fundamental elements: 1-All mature tubes contain a single lumen; 2-Lumen opening requires its filling. Typically, one or more lumens initially form through cellular rearrangements to establish a restricted space. Then, lumens expand through fluid accumulation, common in vertebrates, nematodes, and tunicates, or through matrix secretion, as observed in insects. While fluid and matrix secretion occur through different mechanisms, they function in the same way to support and power luminal expansion.

### Fluid secretion driving lumen opening

The use of fluid secretion during lumen expansion is a key, conserved process in vertebrate organogenesis. In many tubes that undergo cord hollowing, fluid secretion drives expansion of nascent lumens to form a single tube. In zebrafish, the gut tube forms from a solid rod of cells in which TJs are first assembled at multiple actin-rich foci [8]. Then, the activity of the Na^+^/K^+^-ATPase drives the opening of several lumens that begin to coalesce as they expand [9]. Also essential for single lumen formation is the TJ protein Cldn15la, which forms ion-selective pores that allow the accumulation of fluid [9]. Lumen expansion and coalescence are tightly regulated at the transcriptional level by Hnf1b, which controls the expression of Cldn15la and the Na^+^/K^+^-ATPase [9]. Similarly, during development of the zebrafish brain, Claudin5a is required for the establishment of a tight barrier that facilitates fluid accumulation and ventricle inflation [10]. The importance of TJs in lumen opening has also been shown in mammals where they play key roles early in embryogenesis as shown for the mouse blastocyst where Cldn4 and 6 are required for blastocoel opening [11].

**Figure 2:**
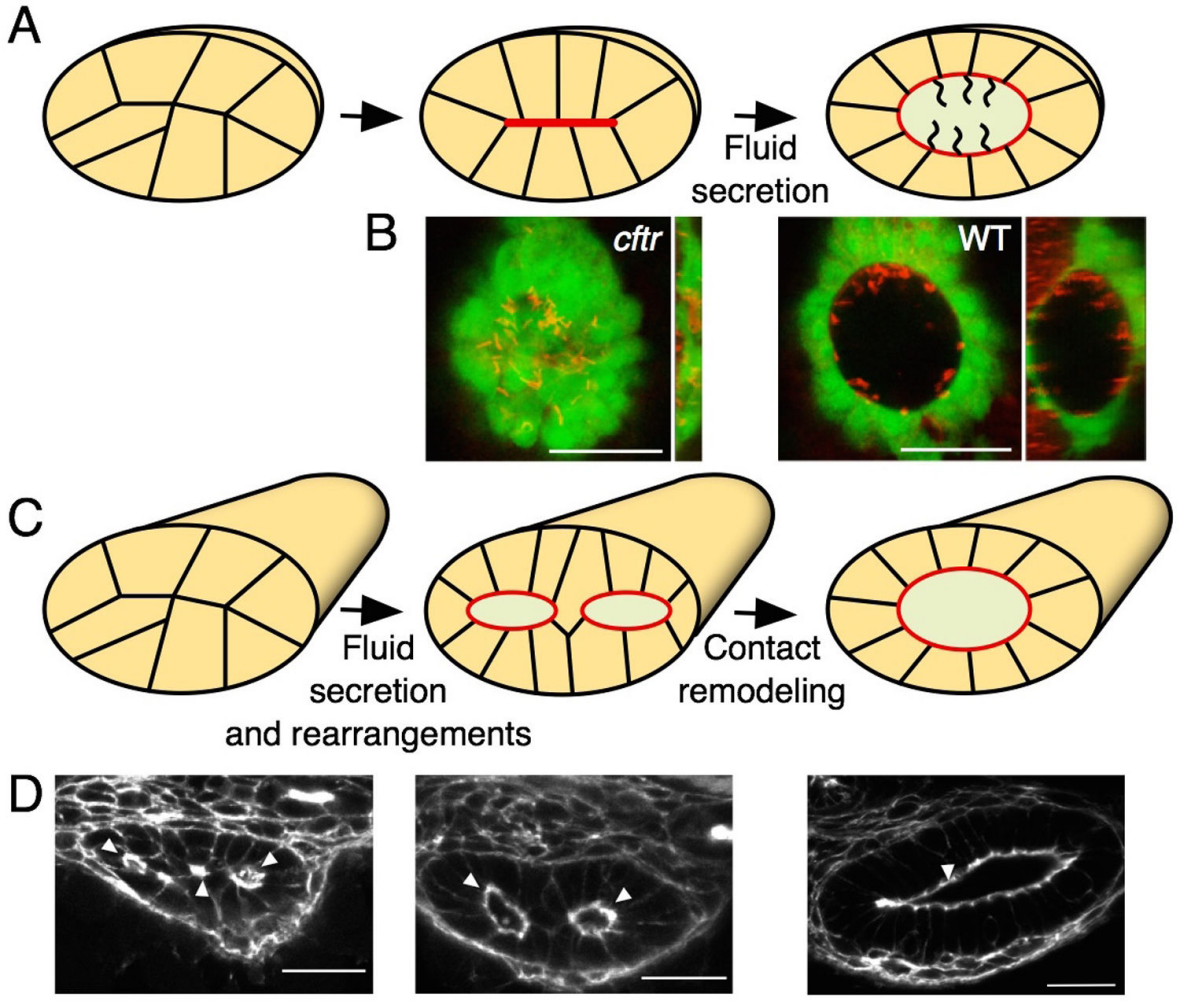
Fluid secretion during lumen formation (A) Schematic representation of Kupffer's vesicle (KV) lumen formation. The KV lumen forms from a group of initially disordered cells. Cellular rearrangements and fluid secretion generate a single central lumen with motile cilia that generate circular luminal fluid flow. (B) The importance of fluid secretion during lumen formation is illustrated by ventral and orthogonal views of the *cftr* mutant zebrafish KV, which contains a central plate of apical membrane and no luminal space, in contrast to the dramatic expansion of the WT organ (adapted from [15]). The KV epithelium (green) is labeled by *sox17:GFP* expression and the cilia are labeled by Arl13b (red). (C) In the zebrafish gut, lumen formation is initially characterized by lumen coalescence and expansion leading to a transient double lumen stage, which is resolved by Rab11-dependent contact remodeling. (D) This process is illustrated by stages of lumen formation in cross sections of the zebrafish gut (adapted from [27]). Filamentous actin (white) is marked by phalloidin.

A key driver of vertebrate fluid secretion is the chloride channel CFTR. This channel regulates fluid secretion in several organs from morphogenesis through out life and its loss of function causes cystic fibrosis [12]. Perhaps the clearest example of how CFTR-dependent fluid secretion functions during lumen opening comes from Kupffer's vesicle (KV), the zebrafish organ of laterality [13]. The KV lumen opens through a process of cord hollowing from several small lumens that expand and merge into a single lumen [14,15]. In KV, loss of Cftr function blocks lumen expansion and organ function [15]. The loss of fluid secretion leads to a complete absence of the luminal space, even though the surrounding epithelial cells develop normally and exhibit proper apico-basal polarity [15] (Figure 2A,B). Interestingly, the lack of a luminal space does not impair the formation of normal motile cilia, indicating that fluid secretion and flow are specified independently. CFTR is similarly required for lumen expansion in the mammalian salivary gland [16].

Fluid-driven lumen expansion has also been observed in other organs. In the zebrafish brain, Na^+^/K^+^-ATPase is essential for ventricle inflation, independent of the circulation required for later stages of expansion [17,18]. Similarly, fluid secretion is required to inflate the zebrafish otic vesicle (see review by T. Whitfield). Hydrostatic pressure is also required for the formation of lumens that traverse single cells. During tube formation in the *Ciona intestinalis* notochord, Slc26-dependent fluid secretion is required for the development of the lumen along the length of the notochord rod [19]. A similar process also occurs during the formation of the *C. elegans* excretory canal. In this organ, aquaporins facilitate the movement of water required for unicellular tube extension [20,21].

### Matrix secretion during lumen formation

Lumen expansion driven by hydrostatic pressure is crucial for lumen expansion in vertebrates, but this is not the only mechanism used by metazoans. In the Drosophila tracheal system, a chitinous matrix is secreted into the lumen, which is later cleared to leave a gas-filled tube [22]. Matrix secretion into the trachea and salivary gland depends on the activity of the secretory pathway regulated by the Creb family transcription factors [23,24]. While matrix secretion serves a similar role as fluid, the forces generated by the matrix can be locally controlled by spatially restricted secretion as clearly shown for the Drosophila hindgut [25]. Apical matrix secretion is also a key component of lumen expansion in other Drosophila organs including eye ommatidia [26] and salivary glands [23]. These examples highlight the critical role of intraluminal forces during lumen formation.

### Lumen coalescence: allied forces drive lumen resolution

An essential step in tube formation is the resolution of a single lumen, which requires coordinated tissue remodeling and luminal expansion. In the zebrafish gut, opening of multiple lumens is followed by a transient double lumen stage whose resolution requires the remodeling of cellular contacts [27] (Figure 2C,D). Loss of mesenchymal *smoothened* does not block fluid-driven lumen expansion, but arrests lumen formation at the double lumen stage due to impaired Rab11-dependent contact remodeling [27]. Similarly, lumen resolution in the mouse salivary gland is also regulated by surrounding tissues [16]. It would be interesting to investigate whether innervation, besides its role in promoting CFTR-dependent secretion, also controls tissue remodeling in this organ.

Contact and TJ rearrangements are also required for single, continuous lumen formation in the *Ciona* notochord and the zebrafish vasculature. During anastomosis of the zebrafish vasculature TJ remodeling and fluid pressure work together to generate a continuous lumen [28]. In some cases, lumen expansion and coalescence also require the generation of a permissive environment. In the zebrafish brain, luminal expansion is restricted by a myosin phosphate regulator, which must relax to allow the lumen to inflate [29]. In the mammalian vasculature, lumen opening requires the loss of apical adhesions mediated by mucin secretion [30].

## Pressing structures into shape

Fluid pressure can influence epithelial folding, cell proliferation, and can exert global effects on a whole organism as seen during the elongation of the AP axis. Classic studies have established that regulated fluid pressure is essential for the normal development of the brain and lung. In the chick brain, fluid pressure inflates the ventricular lumen after the spinal neurocoele becomes occluded [31]. Draining this cerebrospinal fluid to relieve the pressure in the brain leads to dramatic changes in the morphogenesis of the neuroepithelium including extreme folding, reduced cell division, and slowed growth of the neural tissue [32].

Lung morphogenesis is also dependent upon properly regulated fluid pressure. Modulating fluid pressure in the developing lamb lung leads to changes in the growth rate of the organ and in the development of alveoli, suggesting that fluid is a key driver of lung development [33]. Although that study suggested a relationship between fluid pressure and lung development, it will be interesting to examine whether fluid pressure also influences branching.

### Axial development and moving worms

**Figure 3:**
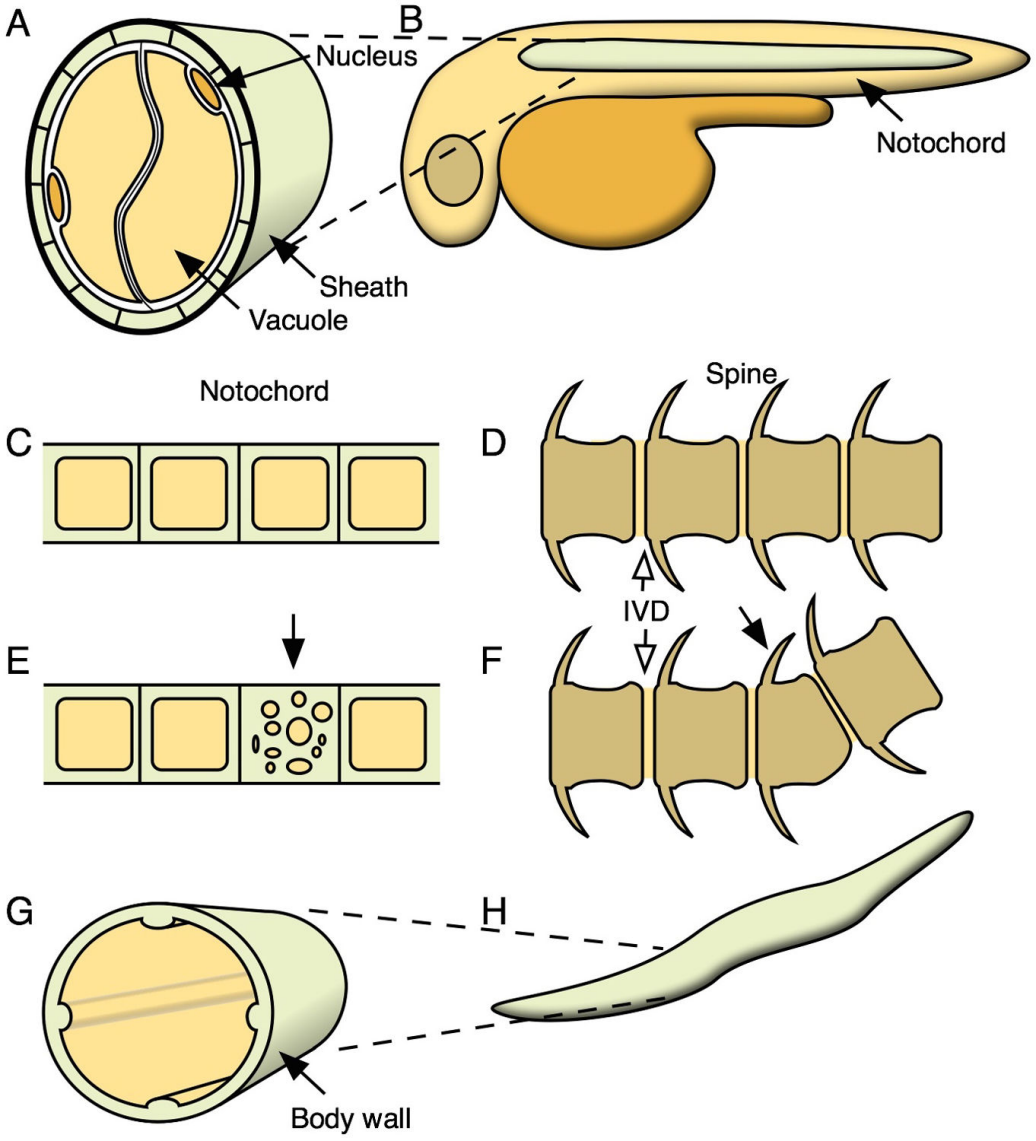
Fluid secretion shapes morphogenesis (A) The notochord is composed of cells containing large fluid filled vacuoles, surrounded by a sheath of extracellular matrix. (B) The hydrostatic pressure from the notochord acts as a hydrostatic skeleton and helps elongate the embryo. (C,D) A properly developed notochord serves as a scaffold for normal vertebral development. (E,F) Disruption of notochord vacuoles can lead to defects in spine morphogenesis and vertebral malformation. For simplicity, the sheath cells are not depicted in C and E. (G,H) The C. elegans body plan depends on internal fluid force restricted by extracellular matrix in the body wall.

Fluid pressure also functions in vertebrate axis elongation, which partly depends on hydrostatic pressure generated within the notochord. The vertebrate notochord is composed of a core of cells that contain large fluid-filled vacuoles enclosed by a sheath of epithelial-like cells surrounded by extracellular matrix [34] (Figure 3A). In a seminal study by Koehl and Keller, dissected frog notochords were shown to be osmotically active and changing the tonicity of the medium altered the rigidity and shape of the notochord rods [35]. Adams et al. also explored the structure of the extracellular matrix and concluded that collagen fibril orientation helps direct the fluid pressure axially. These elegant ex vivo and modeling studies suggested that fluid pressure within the inflating notochord help elongate and straighten the embryonic AP axis (Figure 3B). Intrigued by this work we decided to study the formation and function of the fluid-filled notochord vacuoles in vivo using zebrafish. Cell biological and genetic manipulations indicated that these vacuoles are lysosome related organelles whose inflation and integrity requires post-Golgi trafficking and the activity of the V-H^+^-ATPase [36]. In vivo, loss of vacuole formation or integrity led to embryos with shortened AP axis as previously predicted, but straightening was not impaired. Unexpectedly, when we followed the development of these fish further we found that notochord vacuoles are also required for proper spine formation [36]. Loss of vacuolated cells or mosaic disruption of vacuole integrity caused kinks in the spine axis similar to what is seen in scoliosis patients (Figure 3C-F). Interestingly, defects in sheath matrix components also cause kinking of the spine [37]. Based on our initial and subsequent studies we proposed that the fully inflated vacuoles and their arrangement within the matrix casing not only produces an elongating force along the AP axis, but also resists compression from vertebral bone growth during spine formation [38].

Keller also noted that luminal pressure and a restrictive outer wall may be a common theme for directing organogenesis [39]. In *C. elegans*, the body shape and mechanics of the worm depends on internal fluid pressure restricted by a stiff external cuticle [40-42] (Figure 3G,H). A similar balance of forces may also contribute to the morphogenesis of the *Drosophila* oocyte where deposition of an elaborate matrix is required for generating an elliptical structure [43], but here the role of internal fluid pressure remains to be determined.

## Under Pressure: physiological and pathological effects of fluid secretion

Luminal fluid pressure also acts on the surrounding epithelium. In the mammalian bladder, cyclic inflation and drainage events stretch and relax the epithelial cells. In response, the cells deliver new apical membrane to the luminal surface to relive tension on the epithelium and allow the bladder to expand [44,45]. Once the pressure is relieved, cells can internalize excess membrane and store it for future expansion.

Regulated secretion and absorption is essential for normal organ function and loss of this balance leads to diseases ranging from secretory diarrheas and polycystic kidney disease (PKD) to cystic fibrosis. Pathogens like cholera can activate cyclic AMP signaling, which increases CFTR activity and leads to uncontrolled intestinal fluid secretion. In the human kidney, fluid accumulation within the nephra causes PKD. While PKD is triggered by disruption of ciliary function, CFTR-dependent fluid secretion also contributes to cystic expansion [46,47]. The barrier function is also essential for maintenance of fluid homeostasis and organ function. In the mouse intestine claudin-15 promotes fluid absorption by allowing sodium ions to pass through the tight junctions [48]. Loss of claudin-15 causes defects in fluid absorption and a corresponding increase in intestine size. In the kidney, claudins 2 and 4 facilitate renal fluid absorption [49,50].

Loss of fluid secretion can also lead to human disease. Loss of CFTR activity leads to cystic fibrosis, characterized by mucosal buildup and colonization by opportunistic pathogens in the lung [51]. While loss of CFTR during human development does not lead to severe developmental defects, subtle reductions in the size of lumenal organs like the trachea have been observed [52], likely due to reduction of internal fluid pressure during fetal development.

Regulators of vertebrate fluid secretion have been identified by forward genetic screens in the zebrafish. The *baobab* mutant presents a rapid and dramatic increase in intestinal fluid accumulation and a secretory diarrhea phenotype due to loss of a key negative regulator of Cftr [53]. Thus, forward genetics and modifier screens may help identify mechanisms that regulate fluid homeostasis during development and disease.

## Concluding remarks

The long range forces generated by fluid pressure are key drivers of many morphogenetic processes. The outward force generated by fluid secretion can drive lumen expansion, influence the shape of developing tissues, and provide proliferative cues. Furthermore, as exemplified by the notochord, these forces can exert effects that span and guide development across an entire embryo. Understanding the functions of hydrostatic fluid pressure during morphogenesis is also likely to provide broad insights into the regulation and responses to diseases characterized by misregulated fluid secretion. Fluid forces represent a key morphogenetic process essential for shaping embryos throughout development.
